# Ein weiteres Kleid – Zur Wissensgeschichte häuslich-textiler Umgebungen im 19. Jahrhundert

**DOI:** 10.1007/s00048-020-00290-4

**Published:** 2020-12-14

**Authors:** Kira Jürjens

**Affiliations:** Sprach- und literaturwissenschaftliche Fakultät, Institut für deutsche Literatur, Humboldt-Universität zu Berlin, Unter den Linden 6, 10099 Berlin, Deutschland

**Keywords:** Interieur, Hygiene, Textilien, Gender, Wohnen, Umwelt, Literatur, Interior, Hygiene, Textiles, Gender, Habitation, Environment, Literature

## Abstract

In diesem Artikel wird untersucht, inwiefern Textilien in den einrichtungstheoretischen, medizinisch-hygienischen und literarischen Auseinandersetzungen mit dem Wohnen im 19. Jahrhundert als funktionale Umgebungen des Lebendigen entworfen werden. Damit sind zugleich geschlechtliche Zuschreibungen verbunden, die die Frau als Verantwortliche für die Regulierung dieser textilen Umgebungen ausmachen. Während es so einerseits zu einer naturalisierenden und mechanisierenden Überblendung von Frau und Wohnraum kommt, ist mit der Verwissenschaftlichung und Technisierung des Wohnens andererseits eine weiblich konnotierte Expertise verbunden, die nicht allein auf den Innenraum des Hauses begrenzt ist. Der Artikel nimmt die Verhandlungen textiler Expertise und Gestaltungsmacht in unterschiedlichen Wissensbereichen und Darstellungsformen in den Blick, um das Verhältnis von Textilien, Wohnen und Weiblichkeit im 19. Jahrhundert neu zu perspektivieren.

## Abstract (en)

Drawing on sources such as home furnishing manuals, hygiene and household guidebooks, as well as literary texts, this article examines how the nineteenth-century domestic interior can be regarded as a functional environment regulated by textiles. In the debate about the correct usage of furnishing textiles in line with contemporary aesthetic and sanitary standards, women were held responsible for regulating these textile environments. While on the one hand this led to a supposedly natural and mechanical conflation of women and domestic space which has often been interpreted as restrictive and encapsulating, my aim is to identify a specific feminine agency that has its origin in the home but is not limited to it. By tracing the negotiations of this gendered textile expertise in different fields of knowledge and forms of representation, this article reevaluates the relationship between textiles, domesticity and femininity in the nineteenth century.

Zu Beginn des 20. Jahrhunderts wird die bürgerliche Wohnung voriger Generationen zum Sinnbild weltabgewandter, dysfunktionaler Plüschigkeit und dient als Kontrastfolie, vor der sich Rationalisierungsbestrebungen, Aufbruchsnarrative sowie Konsum- und Gesellschaftskritik einprägsam inszenieren lassen. Mit der sich im ausgehenden 19. Jahrhundert durchsetzenden Bakteriologie wird aus medizinischer Perspektive die Wichtigkeit glatter, waschbarer und keimfreier Oberflächen betont, und hochflorige Stoffe geraten als idealer Nährboden für krankmachende Keime unter Generalverdacht. Vor diesem Hintergrund richtet sich die architektonische Avantgarde mit großem Entkleidungs- und Entrümpelungsgestus gegen die verstaubten Hüllen und Stoffschichten der Vergangenheit. Zwischen mystifizierender Nostalgie und marxistischer Kritik entwirft Walter Benjamin in seinen Beschreibungen der Lebenswelten des 19. Jahrhunderts die Wohnung als ein den Bürger regelrecht versargendes Etui (1972: 89).^1^ Frühe feministische Texte betonen den be- und einschränkenden Charakter des häuslichen Interieurs und verknüpfen Emanzipation und Befreiung mit der Reduktion von Textilien in der Kleidung wie in der Einrichtung.^2^

Dieser Beitrag hat zum Ziel, die imaginationsgeschichtlich bis heute wirksame Verschränkung von Textilien, Einschluss und Weiblichkeit im Interieur wissensgeschichtlich auszudifferenzieren und zu hinterfragen.^3^ Dazu nehme ich Quellen aus ganz verschiedenen Diskursen ab der Mitte des 19. Jahrhunderts in den Blick. Von der Medizin, die noch von Miasmentheorie und aufsteigender Sozialhygiene geprägt ist, über Pädagogik und Ästhetik bis hin zur Literatur werden Fragen des textil geprägten Wohnens in verschiedenen akademischen Fachdisziplinen und Wissensgebieten als Gegenstand aufgegriffen. In Ratgebern, Familienzeitschriften und populären Vorlesungen wird das textile Wohnwissen publikumswirksam verhandelt und verbreitet. Dabei teilen die einrichtungstheoretische, hygienische und literarische Auseinandersetzung nicht nur das Interesse für die Frage nach dem ‚richtigen‘ Wohnen, sondern auch Publikationsorgane wie *Die Gartenlaube*. Anhand exemplarischer Stellenlektüren von Einrichtungs- und Haushaltsratgebern sowie hygienischer und literarischer Texte des 19. Jahrhunderts lässt sich zeigen, dass in der Diskussion um Einrichtungswissen und Haushaltstechnologien die (Wohn‑)Textilien keineswegs bloßer Verpuppung und Abdichtung dienen, sondern in funktionale Austauschbeziehungen zwischen innen und außen eingebunden sind. Damit verbindet sich in den untersuchten Texten auch die Verhandlung von Geschlechterrollen: Mit Wohnungseinrichtung und -ausstattung betraut, gewinnt die Frau im 19. Jahrhundert eine gewisse hygienische, ökonomische und ästhetische Handlungs- und Gestaltungsmacht, was im (populär‑)wissenschaftlichen und literarischen Diskurs wiederum unterschiedliche Zuschreibungen und Erzählungen hervorbringt.

Vor diesem Hintergrund ist, den Ausführungen Amanda Vickerys folgend, die Vorstellung der Frau des mittleren und gehobenen Bürgertums als Gefangener im „upholstered cage“ zu relativieren (1993: 388). Während die sozialen Wechselwirkungen und Repräsentationsverhältnisse zwischen Innen- und Außenraum, die die klare Unterscheidung von privatem Wohnraum und außerhalb liegender Öffentlichkeit hinfällig werden lassen, in der Forschung gut herausgearbeitet wurden (Vickery 1993; Gavison 1992; Peterson 1984), erscheint mir die materiell-funktionale Verflechtung von Umgebung und Bewohnenden im Interieur bisher weniger beleuchtet. So bietet es sich an, die Diskussion um das ‚richtige‘ Wohnen im 19. Jahrhundert als eine besondere Form der in den Lebenswissenschaften geführten Auseinandersetzung mit Umgebungen des Lebendigen zu betrachten. Aus dieser Perspektive wurden in der Forschung bisher vor allem gebaute Architektur und Bauplanung in den Blick genommen (Stalder 2017; Banham 1984; Teyssot 1989). Die an Matratzen, Teppiche oder Vorhänge geknüpften Umgebungstechnologien geraten dabei leicht aus dem Blick, was sicherlich auch mit der geschlechtlichen Konnotation dieser jeweiligen Umgebungen zusammenhängt. Einrichtung und Ausstattung des Hauses lassen sich zu den an den privaten Raum gebundenen und daher tendenziell übersehenen „feminine technologies“ zählen, die, wie Judith McGaw gezeigt hat, zunächst ans Licht gebracht werden müssen (McGaw 2003: 17). Dabei kommt dem Kleid als zentraler Denkfigur eine besondere Rolle zur Veranschaulichung der Wechselwirkung zwischen dem Menschen und seiner materiellen Umgebung zu. In seiner Eigenschaft als Hülle und buchstäblicher Zwischenschicht bietet sich das Kleid als eine Art materielle Konkretion des Milieus (wörtlich ‚mittlerer Ort‘) an, das begriffsgeschichtlich eng mit dem lateinischen ‚ambiens‘, dem Umhüllenden, verwandt ist (Spitzer 1942: 2; Brandstetter & Harrasser 2010: 13; Wessely & Huber 2017: 7).

Mit dieser Fokussierung auf die Textilien des Innenraumes und die daran geknüpften geschlechtlichen Besetzungen schließt der Artikel an die Forschungsdiskussion zu Materialität an, wie sie seit einiger Zeit ausgehend von der Kunst- und Kulturwissenschaft (Wagner & Rübel 2002) und zunehmend auch für die Literaturwissenschaft an Bedeutung gewonnen hat (Vedder & Scholz 2018). Dabei hat sich ein doppelter Zugriff etabliert, der darauf zielt, dargestellte Materialien sowohl in der ihnen je eigenen stofflichen Beschaffenheit und Eigensinnigkeit als auch in ihrer Einbindung in Prozesse der Bedeutungsgenerierung zu untersuchen (vgl. Kalthoff et al. 2016: 25). Dieser Ansatz soll auch für die hier untersuchte Frage nach der Funktionalität textiler Wohnumgebungen und den damit verknüpften Geschlechterrollen übernommen werden. Wenn dabei von Textilität und nicht von Materialität oder Stofflichkeit die Rede ist, dann mit dem Ziel der materiellen Spezifizierung. Die Materialvielfalt des 19. Jahrhunderts als „Saeculum der Dinge“ (Böhme 2006: 17) soll hier auf die besonderen Eigenschaften von Textilien und den Grad ihres Einsatzes zugespitzt werden. Die vor dem Hintergrund veränderter Produktions‑, Distributions- und Konsumtionswege neuartige Textilität des Wohnens im 19. Jahrhundert umfasst Fenster‑, Bett- und Türvorhänge, Stofftapeten, Teppiche, Matratzen, Decken, Polster, Kissen, Tisch- und Zierdecken sowie Schonüberzüge, Putzlappen, Staubtücher und Fliegengitter. Diese Textilien werden nicht nur in ihrer visuellen Wirkung als Dekoration, sondern auch und gerade in ihrer materiell-konkreten Beschaffenheit mit ihrer jeweils spezifischen Faserglätte oder Gewebedichte zum Diskussionsgegenstand in den verschiedenen Wissensbereichen.

Im ersten Teil dieses Beitrags ist grundsätzlich zu zeigen, inwiefern die Wohnung in den unterschiedlichen Diskurszusammenhängen des 19. Jahrhunderts als eine Umgebung des Lebendigen entworfen wird. Darauf aufbauend sollen dann die ästhetische und hygienische Argumentation jeweils näher in den Blick genommen werden. So lässt sich anhand der von Kunsthistorikern wie Jakob von Falke und Cornelius Gurlitt geführten ästhetischen Diskussionen um die geschmackvolle Innenraumausstattung verfolgen, wie diese unter der Hand der Hausfrau zur bildenden Umwelt für die Familienmitglieder wird. Aus der hygienischen Perspektive, die sich in den zahlreichen Haushaltslehrbüchern auch explizit an die Frauen richtet, ist wiederum zu zeigen, dass Textilien nicht allein als unreinliche Staubfänger wahrgenommen wurden, sondern ihnen als gesundheitsfördernde und wärmeregulierende Mittel für die Gesundheit des Hauses zentrale Bedeutung zukam. Besondere Aufmerksamkeit gilt dabei Bett und Schlafzimmer als Zonen erhöhter Textilität.

Neben Hygiene sowie Haushalts- und Einrichtungslehre ist die Literatur ein weiterer wichtiger Ort zur Verhandlung von häuslichem „Umgebungswissen“ (Wessely & Huber 2017: 7). Am Beispiel des österreichischen Autors Adalbert Stifter möchte ich verfolgen, dass textile Interieurs auch in der Literatur nicht allein als dekorative Kulisse oder Gegenstand biedermeierlicher Rückzugsphantasien dienen. Vielmehr werden Textilien in den ab den 1840er Jahren erscheinenden Erzählungen als existenzielle Lebensgrundlage und Mittel zur Regulierung des Verhältnisses von Mensch und Umgebung in narrative Handlungs- und Darstellungszusammenhänge eingebunden, und gewinnen so für ein realistisches, an den materiellen Widerständigkeiten und Durchlässigkeiten interessiertes Schreiben an Bedeutung.

Im Gegensatz zum (populär-)wissenschaftlichen Diskurs der zweiten Jahrhunderthälfte erweist sich die geschlechtliche Zuständigkeitsverteilung hier eher als Gegenstand dynamischer Verhandlungen, denn als unumstößliche Tatsache, was auch damit zusammenhängt, dass die weiblich-textile Handlungs- und Gestaltungsmacht schwer mit der männlich-textuellen Autorität der Erzähler- und Figurensubjekte zu vereinbaren ist. Es geht also nicht darum, die historischen Befunde mit den literarischen Beispielen zu illustrieren oder zu beglaubigen, sondern die literarische Verhandlung von Textilien, Wohnen und Weiblichkeit mit ihren eigenen Darstellungsmöglichkeiten, Assoziations- und Imaginationsräumen in den Blick zu nehmen.

Anhand dieser exemplarischen Schlaglichter auf das in Ästhetik, Haushaltslehre, Hygiene und Literatur verhandelte Wissen um den Gebrauch von Textilien möchte ich argumentieren, dass das Interieur des 19. Jahrhunderts nicht trotz, sondern gerade wegen seiner Textilität als eine funktionale Umwelt und Verhandlungsort weiblicher Agency betrachtet werden kann, die ihren Ursprung im Haus hat, aber durchaus über dieses hinausweist. Dass mit Blick auf die asymmetrische Machtverteilung zwischen häuslichem und außerhäuslichem Raum die gesellschaftliche Wirkkraft von Frauen im 19. Jahrhundert grundsätzlich eingeschränkt ist, bleibt unbestritten. Umso wichtiger ist es, das weiblich konnotierte textile Wissen und die damit verbundenen Handlungsspielräume als solche zu benennen und zu beleuchten.

## Die Wohnung als Umgebung des Lebendigen

Als Einsatzpunkt für die „Debatte um die Bedeutung des Milieus für die Architektur“ macht Laurent Stalder das 19. Jahrhundert aus (2017: 72). Während sich die theoretischen Ursprünge der Verflechtung von Architektur und Biologie bis ins späte 18. Jahrhundert zurückverfolgen lassen, schlagen sich die Erkenntnisse der Milieutheorie – vor allem ab der zweiten Hälfte des 19. Jahrhunderts – im Architekturverständnis nieder, „da ab diesem Zeitpunkt verschiedene Apparaturen in der Architektur eingesetzt werden, die der präzisen Regulierung der unterschiedlichen Ströme von Licht, Luft, Personen oder Flüssigkeiten, die das Milieu ausmachen, dienen“ (Stalder 2017: 72–73). Solche Formen der Regulierung sind nicht allein Gegenstand der bauplanerischen Beschäftigung mit dem Wohnen, sondern sind auch für Fragen der Ausstattung, Einrichtung, Dekoration und Reinigung der Wohnung, wie sie in Gesundheits- und Einrichtungslehre, Haushaltsratgebern und Literatur diskutiert werden, zentral.

Ein frühes Beispiel für den milieutheoretisch geprägten Blick auf die häusliche Umgebung des Menschen findet sich in Goethes Zugabe zu Johann Caspar Lavaters *Physiognomischen Fragmenten *(1775). Darin weitet Goethe das Repräsentationsverhältnis zwischen Charakter und Gesicht auf „Kleider und Hausrath“ aus, und beschreibt eine wechselseitige Beeinflussung von Mensch und Wohnung:

Was den Menschen umgiebt, wirkt nicht allein auf ihn, er wirkt auch wieder zurück auf selbiges, und indem er sich modificieren läßt, modificiert er wieder rings um sich her. […] Die Natur bildet den Menschen, er bildet sich um, und diese Umbildung ist doch wieder natürlich; er, der sich in die große weite Welt gesetzt sieht, umzäunt, ummauert sich eine kleine drein, und staffiert sie aus nach seinem Bilde. (Lavater 2005: 15)

Mit der Betonung der wechselseitigen Beeinflussung hebt sich Goethe hier durchaus von deterministischen Positionen seiner Zeit ab, in denen der Mensch ausschließlich als „Produkt“ seiner Umgebungsbedingungen begriffen wird (vgl. Lehmann 2017: 122). Im Kern scheint hier gewissermaßen bereits die „dialektische Konzeption der Beziehungen zwischen Organismus und Milieu“ (Canguilhem 2009: 240) der Biologie des 19. Jahrhunderts angelegt. Die Aktivität des Menschen betonend, versöhnt Goethe hier Lavaters theologisch orientierte Physiognomie mit zeitgenössischen lebenswissenschaftlichen Positionen und schwächt deren blasphemischen Schock ab, indem er dem Menschen zumindest in seiner „kleine[n]“ Welt gottgleiche Agency („nach seinem Bilde“) zuspricht. Wenn Goethe dabei Umzäunen, Ummauern und Ausstaffieren als Effekte eines ursprünglich natürlichen Impulses darstellt, zeigt sich dennoch die für die Konzeption des Milieus grundsätzliche Verwischung der Grenzen zwischen Körper und Natur sowie Kultur und Technik (Altamirano 2016).

Auch wenn es in dem Zitat vordergründig darum geht, ein Repräsentationsverhältnis zwischen Mensch und Umgebung herzustellen, wird zugleich ein milieutheoretisch geprägter Funktionszusammenhang beschrieben, wie er für die Auseinandersetzung mit dem Wohnen im Verlauf des 19. Jahrhunderts immer wichtiger wird. Es ist diese bei Goethe angedeutete und milieutheoretisch begründete Doppelfunktion, auf die sich auch der später genauer zu untersuchende Aufstieg des Interieurs in der Literatur des 19. Jahrhunderts zurückführen lässt: Die Wohnung ist als Gegenstand kontinuierlicher Gestaltungsarbeit des sich in ihr abbildenden Bewohners und in ihrer Funktion als den Bewohner wiederum umbildenden Umgebung nicht einfach nach visuellen Kriterien gestalteter, dekorativer Hintergrund, sondern in ihrer spezifischen Materialität funktionale Lebensgrundlage der Figuren und erzählerisches Mittel zur Reflexion der eigenen darstellerischen Verfahren. Dabei schafft die Literatur – wie in der Forschung vor allem am Beispiel von Balzac und Zola gezeigt wurde – wiederum derart wirkmächtige und narrativ abgesicherte Begründungszusammenhänge, dass ihr für die Karriere der Milieu- und Umwelttheorien im 19. Jahrhundert besondere Bedeutung zukommt (Wessely & Huber 2017: 9).

Die Annahme einer Wechselwirkung zwischen dem Menschen und seiner Umgebung ist auch für die medizinische Hygiene des 19. Jahrhunderts entscheidend, die die Aufmerksamkeit von kranken Körpern auf potentiell krankmachende bzw. gesundheitsfördernde und krankheitsverhütende Umgebungen verlagert. Die Wohnung wird so zum Gegenstand des medizinischen und im Sinne der Sozialhygiene auch öffentlichen Interesses. Damit geht zugleich eine Umwertung von Textilien einher. Während Stoff seit dem Mittelalter vor allem als optischer Indikator von Sauberkeit galt (Vigarello 1987), dienen Textilien in der modernen Hygiene nicht mehr allein als Darstellungsmittel, sondern auch zur funktionalen Regulierung von Wärme, Licht und Luft (Sarasin 2001: 17). Der französische Mediziner und Hygieniker Jean-Baptiste Fonssagrives arbeitet in seiner umfangreichen Studie zur häuslichen Hygiene, *La Maison *(1871), mit einem Modell der aufeinanderfolgenden Schichten, in deren Mitte („milieu“) der Mensch angesiedelt sei (1871: 1). Diese Schichten reichen vom Kleidungsstück über das Haus und die Stadt bis zum Klima und bilden eine Abfolge an Einflüssen, die auf den Menschen einwirken und zugleich aber auch von ihm selbst gestaltet und verändert werden.^4^ In ihrer Nähe zum Menschen kommt Kleidung und Behausung in diesem Modell entscheidende Bedeutung zu. Dabei werden sie in der gleichrangigen Nennung dem Klima als natürlicher Bedingung angenähert. Zugleich lässt Fonssagrives keinen Zweifel daran, dass besonders die Frau für die gesunde Ausstattung des Hauses verantwortlich ist und etabliert damit das „privilegierte Bündnis von Mutter und Arzt“, auf das Philippe Teyssot hingewiesen hat (1989: 38).

Während die milieutheoretischen Anleihen der hygienischen Auseinandersetzung mit der Wohnung auf der Hand liegen, ist dieser Zusammenhang für die ästhetische Diskussion der Wohnung weniger deutlich. Wenn in der im deutschsprachigen Raum ab den 1870er Jahren von Kunsthistorikern wie Jakob von Falke und Cornelius Gurlitt geführten, ästhetisch orientierten Diskussion um das ‚richtige‘ Wohnen immer wieder eine wesensgemäße und dem Charakter der Bewohner entsprechende Einrichtung gefordert wird, scheint dies vordergründig einem physiognomischen Paradigma verpflichtet, das ein Repräsentationsverhältnis zwischen unsichtbarem ‚Inneren‘ und sichtbarem ‚Äußeren‘ des Menschen voraussetzt. Reduziert man die Auseinandersetzung mit dem geschmackvollen Wohnen jedoch auf Aspekte der Repräsentation, gehen die oben am Beispiel Goethes erläuterten, schon in der Physiognomie angelegten milieutheoretischen Implikationen der Wohndiskussion verloren. Auch die ästhetischen Einrichtungsratgeber entwerfen die Wohnung weniger als rein visuell berechneten Illusionsraum, sondern durchaus als funktionalen Knotenpunkt vielfältiger Austauschprozesse, und machen sich dabei die argumentative Verbindung eines Wechselverhältnisses zwischen lebendigem Organismus und Umgebung zunutze. So kommt es in Cornelius Gurlitts *Im Bürgerhause *(1888) zu einer regelrechten Belebung der Umgebung, wenn für ihn das „wirthlich[e]“ Einrichten von Zimmern bedeute, diese „mit dem Leben der Bewohner [zu] erfüllen.“ (Gurlitt 1888: 5) Im Zuge dieser Belebung folgt auch die Einrichtung einem Entwicklungsprozess – sie solle „am Anfang nicht fertig“ sein, „sondern vollende sich mit dem Leben der Familie“ (Gurlitt 1888: 94). Dabei wird die Kulturtechnik des Einrichtens in biologischen Metaphern naturalisiert, wenn Gurlitt seine Leser_innen auffordert: „Schaffe dir ein eigenes, deinem Wesen entsprechendes Nest und es wird dir gefallen“ (Gurlitt 1888: 4). Mit dieser Naturalisierung des Wohnens verknüpft sich zugleich eine geschlechtliche Besetzung: Wenn Wohnen in Metaphern von ‚Urhütte‘, ‚Nest‘ und ‚dritte Haut‘ naturalisiert wird, erscheint es nur natürlich, dass sich die als Naturwesen imaginierte Frau damit befasst (vgl. Terlinden 2010: 18). Auch wenn sich Gurlitt in der Einleitung für die Beteiligung des Mannes an der Wohnungseinrichtung ausspricht, liegt die Belebung der Wohnung in der Hand der Frau, die den „nüchternste[n] Raum zu einem belebten, durchgeistigten Wesen um[schafft], das Zeugniss von seinen Bewohnern, deren Lebensart und Gemüth ablegt“ (Gurlitt 1888: 4).

Für dieses, in der gesamten Wohn- und Einrichtungsdiskussion sowie in der Literatur des 19. Jahrhunderts präsente Narrativ, das die Frau gleichsam zum Umschaltrelais (Donzelot 1977: 88; Teyssot 1989: 38) zwischen der durchrationalisierten Außenwelt und dem als Rückzugs- und Erholungsort verklärten Innenraum macht, spielen Textilien eine zentrale Rolle (Nierhaus 1999; Rossberg 1999). So kommt dem Bett als textilem Knotenpunkt des Interieurs und „Herrschaftsgebiet der Gattin“ (Gurlitt 1888: 96) besondere Aufmerksamkeit zu:^5^

Die Frau soll die Stätte ihres ehelichen Glückes mit Aufwand ihres vollen Schmucksinnes reich und eigenartig ausstatten. Es ist das Nest, das sie zu bauen hat. Sie wähle selbst die Bettstelle, welche ihr am geeignetsten erscheint, sie kümmere sich um die Ausstattung des Lagers, sie rege die fleissigen Hände, um durch zierliches Nadelwerk dieselbe ganz mit ihrem Wesen zu durchdringen. (Gurlitt 1888: 97)

Im Unterschied zu den im Beitrag von Lisa Malich untersuchten Diskursivierungsprozessen fällt auf, dass der Nestbau hier gerade nicht als instinktive Handlung beschrieben wird, sondern der imperativen Formulierungen („das Nest, das sie zu bauen hat“) bedarf. Wenn sich das „Wesen“ der Frau durch ihre Hände auf das „Nadelwerk“ übertrage, dann ist die Überblendung von Frau und Innenraum aller metaphysischen und naturalisierenden Begrifflichkeit zum Trotz an konkrete Materialien und Techniken gebunden. Die Produktion des ‚Privaten‘ ist harte und mit großem Aufwand zu versteckende Arbeit (Meyer 1982), die allerdings umso unsichtbarer wird, je mehr die Produzentin mit ihrem Gegenstand verschmilzt: Die mechanischen Gesten der Handarbeit verknüpfen sich mit einer Magie der Berührung. Im Zuge dieser Berührungs- und Übertragungslogik wird die Frau gleichsam selbst zur Umgebung.^6^ Entsprechend hält Roland Barthes über die Rolle der Frau, wie sie Jules Michelet um die Jahrhundertmitte beschrieben hat, fest, sie sei „ein an die Menschheit angrenzendes und zugleich außerhalb ihrer liegendes Element, eine Art vollständiger Einfassung des Menschen, kurz ein Milieu“ (Barthes 1980: 173, zitiert nach Teyssot 1989: 29).

Sieht man von der androzentrischen Perspektive solcher Konzeptionen ab, die die Frau auf ihre Eigenschaft des Umgebens reduzieren, ist sie als Milieu und Mittlerin zugleich weniger auf die rein häusliche Sphäre beschränkt, als vielmehr an deren Randzone zu verorten, an der sie die Austauschprozesse zwischen Haus und Außenwelt reguliert. Dementsprechend wird die Wohnung in den Einrichtungstexten auch nicht als abgeschlossenes Gegenstück zur äußeren Umgebung betrachtet, sondern ist stets von dieser durchsetzt. Die den praktischen Einrichtungsratschlägen häufig vorangestellten historischen und geographischen Abrisse unterschiedlicher Wohnstile leiten die Gestaltungsprinzipien zu unterschiedlichen Zeiten und an unterschiedlichen Orten in klimatheoretischer Argumentation her. Jakob von Falke hält in seiner erstmals 1871 erschienenen Abhandlung über die Ausstattung und Dekoration mit dem Titel *Die Kunst im Hause *fest, dass die „klimatischen Einflüsse“ die „größten Verschiedenheiten“ hervorbringen, „die auch ästhetisch von Bedeutung sein müssen“ (Falke 1873: 168). Die Wohnung ist insofern kein der Natur entgegengesetzter Kulturraum, sondern zu einem gewissen Grad immer auch Produkt der umgebenden Natur, die „auf die Anlage und die Anordnung ein[wirkt], auf die Verhältnisse, auf die Art des Schmuckes, die Wahl und die Bestimmung der Farben“ und „sich so auch ästhetische Geltung“ verschaffe (Falke 1873: 168).

Für die Alltagspraxis im Haus heißt das wiederum, dass der permanente Einfluss äußerer Faktoren wie Wetter‑, Licht- und Luftverhältnisse ein ganz eigenes Aufgabenspektrum für die Rolle der Hausfrau mit sich bringt, deren Arbeit sich so als eng mit der Außenwelt verflochten zeigt. Die genauen Anleitungen der Hauswirtschaftslehrerin und Pädagogin Henriette Davidis in ihrem umfassendem Haushaltsratgeber *Die Hausfrau *(1870) zielen immer wieder auf die Regulierung des eng aufeinander bezogenen Verhältnisses von Innen- und Außenraum. So habe sich beispielsweise die Hausfrau beim Lüften der Betten nach der „Witterung“ zu richten (Davidis 1870: 108) und in selten genutzten Räumen sollen die „Rouleaux“ herabgelassen werden, „damit die Farben nicht von der Sonne verbleichen und die Vorhänge nicht vor der Zeit mürbe werden.“ (Davidis 1870: 114) Mit Blick auf die minutiösen Erklärungen für die Pflege und Reinigung der einzelnen Zimmer wird deutlich, dass der Schutz vor äußeren Einflüssen im Innenraum nicht per se durch die gebaute Architektur gegeben ist, sondern an textil geprägte Alltagspraktiken und Techniken geknüpft ist. Noch bevor sich die milieutheoretischen Erkenntnisse in der gebauten Architektur der Wohnmaschinen der Moderne niederschlagen, liegen die buchstäblichen Fäden zur Regulierung des Verhältnisses von Mensch und Atmosphäre in weiblicher Hand.

Während die rhetorische Naturalisierung weiblicher Kulturtechniken einerseits dazu beigetragen hat, die damit verbundene Arbeit unsichtbar zu machen, können andererseits die mit der Vorstellung vom Haus als Umwelt verbundenen milieutheoretischen Implikationen dazu dienen, die weiblich besetzte Arbeit im Haus als Teil zeitgenössisch breit verhandelter und über das Haus hinausreichender Wissenszusammenhänge zu untersuchen.

## Das Interieur als Bildungsort

Mit der Annahme, dass die äußere Umgebung den Menschen bilde, klingt im oben erwähnten Goethe-Zitat implizit eine Vorstellung von Bildung an, wie sie gegen Ende des 18. Jahrhunderts aufkommt und auch für die Konzeption des Interieurs als einflussreicher Umgebung im 19. Jahrhundert zentral bleibt. In Analogie zu biologischen Formgebungsprozessen erscheint die Bildung des Einzelnen entscheidend von den „äußeren Lebensumstände[n] und Einflüsse[n]“ geprägt (Zirfas 2016: 77). Damit verbindet sich eine Konzeption von ästhetischer Bildung, die nicht „allein auf die Auseinandersetzung mit Produkten der sogenannten Hochkultur ging, sondern auch die alltägliche Lebensform und den in ihr sich manifestierenden Persönlichkeitsstil einschloss“ (Ulrichs 2010: 6).

Wenn in dieser Logik auch der Wohnung bildende Funktion zukommt, gewinnt die mit ihrer Ausstattung und Gestaltung betraute Frau entscheidende Wirkmacht. So heißt es in einem 1855 erschienenen Artikel *Über Frauenbestimmung *in der Familienzeitschrift *Die Gartenlaube*, dass die Frau unter anderem „[d]urch ihren feinen Sinn für Anordnung und Ausschmückung des Einzelnen“ ein „Behagen zu verbreiten“ wisse, das auf den „ganzen [Familien]Kreis namentlich aber das Haupt desselben, den Mann, so angenehm, anregend und zugleich beruhigend, ja, man kann sagen, bildend und veredelnd einwirkt“ (Biedermann 1855: 182–183). Dieser durch die Wohnung vermittelte ‚bildende‘ Einfluss der Frau auf die Familienmitglieder wird im Einrichtungsratgeber *Die Kunst im Hause *des Kunsthistorikers und späteren Direktors des österreichischen Kunstgewerbemuseums, Jakob von Falke, pädagogisch zugespitzt. So spricht er in einem eigenen Kapitel zum *Beruf der Frauen zur Beförderung des Schönen* von der „erzieherischen Kraft“ der Wohnungseinrichtung (Falke 1873: 351). Für Falke fallen die als „Kunst im Hause“ zusammengefassten Aktivitäten, wie die Einrichtung und Ausstattung der Wohnung mit Handarbeiten sowie die Anpassung des eigenen Erscheinungsbildes an die Umgebung, in den Aufgabenbereich der Frau. Dabei reflektiert Falke durchaus das Paradox des eigenen Genres, wenn doch die Frau angeblich über ein „angeborenes Talent“ in diesen Dingen verfüge (1873: 351). Ihm zufolge ist der vermeintlich „angeborene Schönheitssinn“ nur „eine Anlage, die unter dem Einfluß äußerer Anregungen erzogen und gebildet werden muß“ (Falke 1873: 351). In einem durchaus modernen Twist betrachtet Falke das Einrichtungstalent der Frau weniger als natürlich bedingt, denn kulturell erlernt, wenn er den weiblichen Vorsprung in Geschmacksfragen als „Resultat der Uebung und der Bildung“ darauf zurückführt, „daß die Fragen des Geschmackes im täglichen Leben überaus zahlreicher an die Frau herantreten als an den Mann“ (Falke 1873: 351).

Wie Falke im Einzelnen genauer darlegt, handelt es sich bei der in den Aufgabenbereich der Frau fallenden „Kunst im Hause“ besonders um textile Arbeiten, wie Stickereien und Möbelstoff- oder Kleiderwahl. Dass der Frau mit ihrer Betätigung auf diesem Feld eine bestimmte erzieherische Verantwortung zukommt, hängt eng mit der milieutheoretischen Konzeption der Wohnumgebung zusammen. So spricht sich Falke für die ästhetische Gestaltung der Wohnung aus, da

es ja diese Gegenstände sind, unter denen wir aufwachsen, von denen wir in frühester Kindheit die ersten Eindrücke und Anregungen zur Bildung unseres Schönheitssinnes empfangen. Auf sie fällt der erste Blick des Kindes, an sie gewöhnt sich sein Auge; mit diesen Gegenständen seiner Umgebung, die ihm zur Erfahrung geworden sind, wird es die neuen und fremden Eindrücke messen und beurtheilen. (Falke 1873: 350)

An die geschmackvoll gestaltete Einrichtung ist so zugleich ein pädagogischer Auftrag für die einrichtenden Frauen gebunden: Die vorbildliche Innenraumgestaltung dient der ästhetischen Bildung der Kinder des Hauses. Eine solche Konzeption des Wohnraums als Vermittlungsort ästhetischen Wissens findet sich auch in den Schriften des Architekten und Kunsthistorikers Cornelius Gurlitt, der einen Lerneffekt für denjenigen ausmacht, der seine Wohnung selbst und „ohne Hilfe von Fachleuten oder der Mode“ ausgestattet und so „mehr gelernt [hat], als er bei hundertfachem Besuch der Museen heimzuschleppen vermag“ (Gurlitt 1888: 99). Wie Falke betrachtet auch Gurlitt die Einrichtungskunst als ernstzunehmenden Wissensgegenstand und stellt dabei ein regelrechtes Konkurrenzverhältnis zwischen der häuslichen Wohnung und der Institution des öffentlichen Museums her.

Ganz im Sinne solcher Annäherungen der Wohnung an eine öffentliche Institution gewinnt die Einrichtung als Aufgabe der Frau auch bei Falke weit über den engeren Familienkreis hinausgehende Bedeutung:

Die Frau, indem sie das Schöne im Hause fördert und ihren eigenen Geschmack und den ihrer Familie und ihrer Umgebung bildet, arbeitet mit an der Bildung der Nation, an der Cultur der Menschheit. (Falke 1873: 351)

Über die Familie hinaus wird der Einrichtungsarbeit so nicht allein nationale, sondern geradezu „civilisatorische Bedeutung“ verliehen (Falke 1873: 351). Darin klingt sowohl die zeitgenössische ideologische Überhöhung der Familie an als auch der eher pragmatisch-hygienische Ansatz den Ursprung nationaler Gesundheit in der Wohnung jedes einzelnen zu suchen. Die damit verbundene weibliche Wirksamkeit wird in konservativen Publikationen rhetorisch eingehegt, wenn zum Beispiel Wilhelm Heinrich Riehl 1855 im dritten, *Die Familie *betreffenden Band seiner umfangreichen *Naturgeschichte des deutschen Volkes *die „Geltung der Frauen im öffentlichen Leben“ als eine „bloß indirekte, in der Familie vermittelte“ beschreibt (Riehl 1861: 98). Ganz im Sinne dieser Einschränkungsrhetorik dient das Umweltkonzept in der Entwicklungspsychologie des 20. Jahrhunderts dann, wie Susanne Schmidt in ihrem Beitrag zeigt, vor allem dazu, eine passiv-statische Vorstellung von Weiblichkeit und Care-Arbeit zu installieren, mit der die selbst ohne Entwicklungspotential entworfene Frau möglichst günstige Bedingungen für die Entfaltung von Mann und Kindern schafft. Diese Argumentation ist gewissermaßen in den Einrichtungsratgebern, die die Frau ja nicht nur als Gestalterin der Umgebung, sondern auch als Teil dieser begreifen, angelegt. Dennoch möchte ich hier für das 19. Jahrhundert zunächst hervorzuheben, dass Autoren wie Falke in ihren Wohntexten durchaus eine milieutheoretisch fundierte Aufwertung der feminisierten Einrichtungs- und Handarbeit vornehmen, mit der die Frau eine nicht allein biologisch begründete über das Haus hinausreichende Wirksamkeit im Rahmen von lehr- und lernbaren Gestaltungstechniken erlangt.

In der Konzeption des Wohnraums als Bildungsort gewinnt dessen geschmackvolle Ausstattung einen über das rein Dekorative hinausgehenden Nutzen. Insofern ist die Vorstellung vom Interieur als privatem Illusionsraum und klar abgegrenztem Gegenstück zu einer rationalistisch organisierten Außenwelt nicht allein mit Blick auf die ökonomischen und sozialen Verwicklungen des Hauses mit dem öffentlichen Leben zu relativieren. Auch die ästhetischen Aspekte der Wohnungseinrichtung sind einem Nützlichkeitsimperativ unterstellt, der die Vorstellung von der reinen Selbstbezüglichkeit des Innenraums infrage stellt.

## Haut – Haus – Kleid: Die hygienische Gleichung

Der Schweizer Architekt Le Corbusier macht 1925 in *L’Art décoratif d’aujourd’hui *kurzen Prozess mit dem textilen Interieur. Darin formuliert er das für die weißen Wände der modernen Architektur wegweisende „Gesetz des Ripolins“, das „jede[n] Bürger“ dazu anhält, „seine Behänge, Damaststoffe, Tapeten, Schablonen durch einen reinen Anstrich von weißem Ripolin zu ersetzen“ (Le Corbusier 1925: 191). An die Stelle der weichen Stoffe mit ihrer jeweiligen Textur und Durchlässigkeit soll mit dem Ripolin, einer 1898 entwickelten weißen Lackfarbe, eine glatte, harte Oberfläche treten. Damit verknüpft ist die Aufforderung in allen Winkeln gründlich zu reinigen: Wohntextilien geraten um die Jahrhundertwende als Sinnbild des Unreinlichen und Unehrlichen in Verruf, was sich bis heute auch in den möglichst hellen und glatten Oberflächen des Krankenhauses niederschlägt, auf die Käthe von Bose in ihrem Beitrag näher eingeht. Dass das ‚Whitewashing‘ der Moderne auch als eine Entfernung von Weiblichkeit gedeutet werden kann, wird mit Blick auf Adolf Loos Aussagen zum Ornament deutlich, der dieses mit dem Weiblichen, Wilden und Erotischen assoziiert (Loos 1962: 175, vgl. dazu auch Nierhaus 1999: 92 und Geiger 2003: 67).

Die Architektur orientiert sich damit an den medizinisch-bakteriologischen Erkenntnissen des ausgehenden 19. Jahrhunderts: Mit Robert Kochs Entdeckung der Bakterien als Krankheitsauslöser entscheidet sich die im 19. Jahrhundert geführte Debatte um die gesundheitsfördernde oder -schädigende Wirkung von Textilien gegen den Stoff, der als potentieller Keimträger endgültig verdächtig wird. Gleichzeitig bleiben die visuellen Codes der modernen Hygiene und Architektur mit ihren glatten, gereinigten, weißen Oberflächen, wie Mark Wigley gezeigt hat, weiterhin einer letztlich textilen Logik verpflichtet (1995: 5). Das von der Dekoration befreite, vermeintlich nackte Gebäude bleibt mit der weißen Farbschicht bekleidet: „No matter how thin the coat of paint is, it is still a coat.“ (Wigley 1995: xviii).

Über die imaginationsgeschichtlich wirksame Assoziation von Stoff mit Unreinlichkeit gerät das andere Lager der hygienischen Debatte des 19. Jahrhunderts leicht aus dem Blick. So spielen Textilien als Mittel zur Regulation von Luft‑, Licht- und Wärmeverhältnissen bei den Vertretern der Miasmentheorie für die gesundheitsfördernde bzw. -erhaltende Funktion der Wohnung eine zentrale Rolle. In den Schriften des Begründers der wissenschaftlichen Hygiene in Deutschland, Max von Pettenkofer, kommt Kleidung Modellcharakter zu, um die Notwendigkeit poröser Wände für den Lufthaushalt der Wohnung zu verdeutlichen. In seinen 1872 in Dresden gehaltenen populären Vorlesungen zu den *Beziehungen der Luft zu Kleidung, Wohnung und Boden* heißt es:

Im Ganzen verfolgt das Haus die nämlichen Zwecke wie die Kleidung, es hat den Verkehr mit der uns umgebenden Atmosphäre beständig zu regeln. Nie darf das Haus eine Vorrichtung sein, uns von der äussern Luft abzuschliessen, so wenig als die Kleidung. (Pettenkofer 1873: 39)

Pettenkofers Vergleich von Haus und Kleidung greift implizit die bis in die Architekturtheorie der Frührenaissance zurückreichende Bekleidungsmetapher auf. Bezieht sich diese Metapher traditionellerweise auf den Unterschied von primärer Konstruktion und bekleidender Dekoration (Oechslin 1994: 44), wird diese Vorstellung vom Architekten und Kunsttheoretiker Gottfried Semper Mitte des 19. Jahrhunderts entscheidend modifiziert, indem er alles Bauen auf textile Techniken zurückführt (vgl. von Arburg 2008). Diese bei Semper auch etymologisch begründete Identifikation von ‚Wand‘ und ‚Gewand‘ dient Pettenkofer wiederum für seine physiologische Argumentation (Ekici: 2016), indem er Haus und Kleidung für die Regulierung der Luftzirkulation verantwortlich macht. Dadurch kommt ihnen eine ähnliche Funktion zu, wie der Haut, wie Pettenkofer in einem Artikel von 1851 näher beschreibt. So sei es notwendig,

daß sich der Mensch mit Wänden, die für die Luft bis zu einem gewissen Grade durchdringlich sind, umgebe, wenn er behaglich und gesund wohnen will, als er sich nur in solche Stoffe zweckmäßig kleidet, welche der Luft Durchgang gestatten. Die Poren unserer Wände können ebenso von Wichtigkeit seyn, wie die Poren der Oberhaut unseres Körpers – wie die Poren in der Kalkschale des Vogeleies. (Pettenkofer 1851: 422)

Mit der Analogie von Haus bzw. Wand und Kleid sowie Haut und Eierschale verschwimmen die Grenzen zwischen kulturellem Artefakt und organischer Schutzschicht. Damit greift Pettenkofer zugleich auf die für die Milieutheorie zentrale, bis in die Antike zurückreichende Eierschalentheorie zurück, nach der Himmel, Erde und Elemente wie ein Ei in der Schale aufgebaut seien (Spitzer 1942: 6, 19). Die über diese Analogien veranschaulichte existentielle Dimension von Wohnung und Kleidung machen sich auch die einrichtungstheoretischen Texte zunutze, wenn sie von der Wohnung als „weiteres“ (Falke 1873: 2) oder „weitestes“ (Alsberg 1883: 16) Kleid sprechen.

Pettenkofers an ein breites Publikum gerichtete Vorlesungen arbeiten sich der Reihe nach vor: von der körpernahen Kleidung über das Verhalten der Luft in den Wohnräumen bis hin zu Fragen der Belüftung des Bodens. Dabei bauen die Vorlesungen eng aufeinander auf: In der ersten Vorlesung legt Pettenkofer das in Versuchen genau quantifizierte Luft- und Wärmeverhalten von Kleidung und Textilien dar. Die zitierte Kleid-Analogie bildet insofern das argumentative Scharnier, um die Erkenntnisse der ersten Vorlesung für das Prinzip der atmenden Wände in der zweiten Vorlesung nutzbar zu machen (Pettenkofer 1873; vgl. auch Ekici: 2016).

Pettenkofer sieht dabei von den kulturellen, sozialen und ästhetischen Aspekten der Kleidung ab und hebt stattdessen die physiologische Funktion von Stoffen hervor. Wenn er von Kleidern als „Apparate[n]“ spricht, deren Aufgabe es gerade nicht sei, „die Luft von uns abzuhalten“, sondern vielmehr eine „beständige Ventilation unserer Körperoberfläche“ zuzulassen, wird die Kleidung maschinisiert (Pettenkofer 1873: 25). Entsprechend vergleicht er sie im weiteren Verlauf auch mit „einer calorischen Maschine oder einem Ofen […], der von der Abhitze unserer Körpermaschine geheizt wird“ (Pettenkofer 1873: 26). Kleidung erscheint insofern nicht als feste und abschließende Grenze, sondern als eine Erweiterung des selbst wiederum als Maschine imaginierten Körpers (vgl. Sarasin 2001) und befindet sich mit diesem wie der außenliegenden Luft in stetem Austausch. So ziehe durch unsere Kleider „beständig ein Luftstrom, dessen Grösse abhängig ist – wie bei jeder Ventilation – von der Grösse der Oeffnungen, von der Größe der Temperaturdifferenz zwischen innen und aussen, und von der Geschwindigkeit der uns umgebenden Luft“ (Pettenkofer 1873: 26). Die Kleidung hat insofern Anteil an der „Verwaltung der Wärmeökonomie“ (Pettenkofer 1873: 21), womit die Frage nach dem richtigen Maß des Textilen verbunden ist. Pettenkofer spricht sich, ökonomisch argumentierend, deutlich gegen die Theorie der textilreduzierten Abhärtung des Körpers aus.^7^ Kleider seien „jeder überflüssigen Abhärtung vorzuziehen“, da es „geradezu schädlich“ sei, „sich abzunützen“ (Pettenkofer 1873: 21). Was das für die Einrichtung mit Textilien heißen kann, hat wiederum Fonssagrives in *La Maison *in ganz ähnlicher Begrifflichkeit gezeigt, wenn er sich für die Türvorhänge, die sogenannten Portieren ausspricht. Die Türvorhänge fungieren als bewegliche Form der Doppeltüren, indem sie eine Luftschicht bereithalten, „qui s’oppose à la déperdition du calorique intérieur de la chambre et en conservent par conséquent la température“ (Fonssagrives 1871: 143). Die aufwendig drapierten und mit Troddeln und Fransen verzierten Portieren sind insofern weit mehr als bloßer Teil der textilen Überspannung und Einkapselung, sondern wärmeregulierende Technologie.

Das Argument der Wärmeökonomie greift Pettenkofer auch gegen Ende der ersten Vorlesung noch einmal auf, wenn er seine Ausführungen mit Bezug auf die Ereignisse des deutsch-französischen Krieges um eine nationale Dimension ergänzt und die Wichtigkeit der „Verpflegung“ von Soldaten mit „Kleidungsmitteln“ hervorhebt (Pettenkofer 1873: 35). In kolonialistischer Kriegsmetaphorik beschreibt Pettenkofer die Kleider im weiteren Verlauf als „Waffen“, „mit denen der civilisierte Mensch seinen Kampf gegen die Atmosphäre kämpft, so weit sie ihm feindlich ist, mit denen er sich sein Element, den Luftkreis unterthan macht“ (Pettenkofer 1873: 35). Diese kämpferische Sprache ist weit entfernt von früheren ökologischen Harmonievorstellungen (vgl. Sprenger 2019: 53) und traditionellen Assoziationen des Textilen mit weicher Behaglichkeit: Die Umgebung des Menschen wird zur Kampf- statt zur Komfortzone. Welche Auswirkungen diese Konzeption des Textilen als aktiv an der Regulierung des Wärmehaushalts beteiligtem Mechanismus für die Einrichtung hat, soll nun mit besonderem Fokus auf das Schlafzimmer näher gezeigt werden.

## Schlafkleid und Bekleidungsapparat

In der hygienischen Diskussion um das richtige Wohnen kommt der Gestaltung und Ausstattung des Schlafzimmers besondere Aufmerksamkeit zu. Jean-Baptiste Fonssagrives macht das Schlafzimmer als den Knotenpunkt der häuslichen Hygiene („nœud de l’hygiène domestique“) aus (Fonssagrives 1871: 99). Als Schauplatz von Sex, Geburt, Krankheit und Tod ist das Schlafzimmer auf besondere Weise mit dem Leben und seinen Bedingungen verknüpft und bietet als Zone erhöhter Textilität besonderen Diskussionsstoff. Dass es sich beim Bett bis heute um einen Hotspot der hygienischen Auseinandersetzung handelt, macht die von Käthe von Bose in ihrem Beitrag dargelegte Uneinigkeit des Krankenhauspersonals über die Zuständigkeit für den Bettwäschewechsel und die damit verbundene Berührungsangst deutlich. Haushaltslehrbücher und -ratgeber sowie einzelne Artikel behandeln ab der Mitte des 19. Jahrhunderts nicht nur aus welchen Materialien Matratze, Decken und Kissen bestehen sollten, sondern geben auch detaillierte Anleitungen zur täglichen Pflege und Reinigung derselben (Keil 1868). Wie schon mit Blick auf die ästhetischen Schriften festgehalten wurde, kommt es auch mit den zahlreichen, an die weibliche Leserschaft gerichteten Publikationen zur hygienischen Gestaltung des Wohnraums zu einer „Verwissenschaftlichung der Hausfrauentätigkeit“, die zumindest für Frauen des Bürgertums mit einem Zugewinn an persönlichem Sinn, familialer Macht und sozialem Status verbunden ist (Frevert 1985: 435; vgl. auch Breuss 1999).^8^

Entscheidende Faktoren für die Ausstattung von Schlafzimmer und Bett sind wie auch für die anderen Räume des Hauses vor allem Reinlichkeit und gute Belüftung. So wird immer wieder vor der Platzierung von Betten in Alkoven und Nischen sowie vor Bettvorhängen und Himmelbetten gewarnt, da mit diesen eine gute Belüftung nicht gegeben sei (Gurlitt 1888: 99; Clima 1876: 29).

Wie für die gesamte Wohnung, gewinnt auch in Bezug auf das Bett der Vergleich mit der Kleidung an Bedeutung.^9^ Pettenkofer bezeichnet das Bett als „Kleidungsstück“ oder „Schlafkleid“ (Pettenkofer 1873: 34). Diese Analogie begründet er zunächst materiell, da das Bett aus „denselben Stoffen gemacht“ sei, „wie die Kleidung des Tages, aus Leinwand, Seide oder Baumwolle, die Schichten welche uns zunächst umgeben, dann auch thierischen Fasern, Pferdehaaren oder Federn, wollenen Decken etc. die entfernteren Schichten“ (Pettenkofer 1873: 34). Zugleich ermöglicht diese Analogie auch die Übertragung der zuvor dargelegten apparativen Eigenschaften der Kleidung, wenn Pettenkofer das Bett als textilen Alltagsgegenstand zu einem „Bekleidungsapparat“ technisiert (Pettenkofer 1873: 34). Das Bett übernehme im Schlaf Aufgaben, die der Körper im Wachzustand selbst leistet und gewinnt so geradezu prothetische Funktion:

Die Bettwärme hält auch ohne grösseren Stoffumsatz, bei geringer Wärmeproduction und vollständiger Ruhe den peripheren Kreislauf in der Haut auf einer bestimmten Höhe, und entlastet dadurch die inneren Organe, sie ruhen auf diese Art gleichsam aus. Das Bett ist daher ein höchst wichtiger Apparat für unseren Wärme- und Bluthaushalt. (Pettenkofer 1873: 34)

Während das Bett so einerseits Körperfunktionen übernimmt und als Erweiterung des Körpers wie seiner natürlichen Vorgänge erscheint,^10^ wird es andererseits als „Apparat“ deutlich dem Bereich der Technik zugeordnet. Die für die moderne Architektur grundlegende Vorstellung des Gebäudes als Prothese des Menschen (Wigley 1991) zeigt sich hier auch im mobiliaren Nahbereich und fällt damit nicht in das Aufgabenfeld des männlichen Architekten, sondern erweist sich als von der Hausfrau bediente Maschine. An die Stelle der Intimität des Bettes tritt Funktionalität. Die sich in der zweiten Hälfte des 19. Jahrhunderts verbreitende Tendenz „aus der Wohnung einen simplen Mechanismus zu machen“ (Teyssot 1989: 52), ist insofern nicht auf die architektonischen *hard facts* wie den Umgang mit den neuen Baumaterialien Eisen, Stahl und Glas sowie Wasser- und Stromleitungen begrenzt. Auch die Textilien des Hauses werden einer solchen Technisierung unterzogen und sind insofern nicht Gegenstück, sondern entscheidender Beitrag zur Konzeption der häuslichen Umwelt als Maschine.

Neben der eher an der Miasmentheorie orientierten Sorge um die richtige Belüftung zur Wärmeregulierung ist mit der Frage nach dem richtigen Grad der Textilität im Schlafzimmer auch die von Kontagien als Krankheitsauslösern ausgehende Sorge vor Ansteckung verbunden. Dies ist gleichsam die Kehrseite der wärmeregulierenden und darin gesundheitsfördernden Textilien. Vor allem medizinische Ratgeber richten große Aufmerksamkeit auf das richtige Lüften der Betten und die Reinigung des Schlafzimmers. Schließlich könne das Bett, wie die österreichische Ärztin Anna Fischer-Dückelmann in ihrem 1901 erstmals erschienenen Ratgeber *Die Frau als Hausärztin *festhält, mehr als dies für die Kleider gilt, „Träger von Krankheitsstoffen und ein Sammelort für die Ausscheidungsstoffe des menschlichen Körpers werden“, da sie seltener gelüftet würden „als unsere Kleidung, die mit uns ins Freie getragen wird, und weil sie niemals Gelegenheit haben, sich ohne uns mit reiner Luft anzufüllen“ (Fischer-Dückelmann 1911: 166). Ebenso solle man laut Friedrich Dornblüth auf „Polsterstühle“, „Polstersophas etc.“ sowie „[w]ollene Fenster- und Thürvorhänge“ im Schlafzimmer verzichten, da diese „neben den Betten und Kleidern, vorzugsweise als Schlupfwinkel und Sammelstätten der Ansteckungsstoffe“ dienen, wenn das Schlafzimmer als Krankenzimmer genutzt werde (Dornblüth 1878: 657). Als Ort der Krankheit wird das Schlafzimmer auch zum potentiellen Umschlagplatz für Ansteckungen.

Statt einer Pauschalverurteilung von Textilien als unreinlichen Krankheitsüberträgern, prägt die hygienischen Haushaltslehrbücher ein differenzierter Blick auf Stoffe in ihrer Doppelrolle als Bedingung und Bedrohung körperlicher Gesundheit. Die Wiener Autorin und Haushaltungslehrerin Marie Clima wägt in ihrer auflagenstarken *Haushaltungskunde* (Clima 1876) die unterschiedlichen Stoffarten mit materialkundlicher Expertise je nach ihrer jeweiligen Aufnahmefähigkeit von Staub gegeneinander ab. So nehme zum Beispiel die Leinwand „ihres glatten drallen Fadens wegen den Staub viel weniger an, als das kantige faserige Garn der Baumwollpflanze“ (Clima 1876: 97). Max von Pettenkofer hebt die besondere Eignung des Leinens für den geregelten Austausch von Luft und Flüssigkeit hervor, da es „Wärme und Wasser, wie sie abfliessen, viel besser von der Hautoberfläche weg[nehmen]“ könne und es „weiteren Schichten zu weiterer gleichmässiger Verarbeitung und Ableitung“ übergebe (Pettenkofer 1873: 33).

Auch die zentrale Funktion von Textilien für die Reinigungsarbeit fordert die Assoziationskette von Stoff, Unreinheit und Krankheit heraus. So geht mit den in den Haushaltslehrbüchern genau beschriebenen Reinigungsprozessen auch eine Multiplikation von Textilien einher. Henriette Davidis empfiehlt mehrere farblich unterschiedene Lappen für die einzelnen Zimmer und Arbeitsschritte, „lange weiße baumwollene Armhandschuhe mit Fingern“, eine spezielle „im Schlafzimmer hängende, weiße Bettschürze mit daran befindlichem Latz“ für das Dienstmädchen, um die Betten beim Auslüften nicht zu beschmutzen (1870: 108) sowie „Ueberziehärmel, welche an der Hand zugeknöpft werden und bis über die Schultern reichen“, um die Ärmel des Kleides beim Reinigen der Ofenröhre nicht zu beschmutzen (1870: 148). Die Textilien wirken dabei in doppelte Richtung: Einerseits dienen sie dazu, die Kleidung des Personals vor Verunreinigung zu schützen, andererseits ziehen sie eine Schutzschicht zwischen der Hausangestellten als einem potentiell die Ordnung im Hause gefährdenden Fremdkörper und den häuslichen Oberflächen ein.

Textilien kommt so insgesamt ein ambivalenter Status zu: Während sie einerseits als Erweiterung und Ersatz der Körperfunktionen den Wärmehaushalt regulieren, der Herstellung und Bewahrung von Reinheit dienen und essentieller Teil der menschlichen Umgebung sind, bilden sie umgekehrt auch eine Umgebung, die die Ansammlung von Staub und darin vermuteten Krankheitserregern begünstigt und so als gefährdend bewertet wird. Mit der hygienischen Aufmerksamkeit für häusliche Reinlichkeit geht eine Umwertung von textiler Arbeit einher, die nicht allein als mythopoetisch fundiertes, moralisch und emotional besetztes, natürlich-weibliches Beschäftigungsfeld erscheint, sondern auch zum Gegenstand der wissenschaftlichen Auseinandersetzung mit der Regulierung und Organisation menschlicher Umgebungen wird. Wenn die Expertinnen dieses textilen Umgebungswissens weiblich sind, ist damit zugleich eine Destabilisierung männlicher Autorität verbunden, die weniger in den pragmatischen Ratgebern Platz findet, als in der Literatur.

## Stoff für Lebensgeschichten

Während die präskriptiven Regelwerke der Hygiene, Einrichtungs- und Haushaltslehre sich bemühen, eindeutige Antworten auf die Frage nach dem ‚richtigen‘ Wohnen zu formulieren, werden in den narrativen Imaginations- und Möglichkeitsräumen der Literatur die im 19. Jahrhundert neu zu justierenden Zusammenhänge von Wohnung, Gesundheit, Geschmack und Geschlecht in ihrer ganzen Dynamik und Ambivalenz verhandelt. Noch vor den untersuchten (populär-)wissenschaftlichen Texten des letzten Jahrhundertdrittels arbeitet sich der österreichische Autor Adalbert Stifter in seinen ab den 1840er Jahren erscheinenden Erzählungen an der lebensbedingenden, (charakter-)bildenden und darstellungstechnischen Funktion von textil regulierten räumlichen Strukturen ab.^11^ Damit verknüpft sich ein an den sichtbaren und funktionalen Oberflächen orientiertes ‚realistisches‘ Schreiben und Erzählen: Das Leben von Stifters Protagonisten entfaltet sich nicht mehr wie im klassischen Bildungsroman im Sinne einer ‚inneren‘ teleologischen Entwicklung, sondern im ‚äußerlichen‘ Umgang mit in die Fläche gebreiteten räumlich-materiellen Arrangements.

Auch wenn Stifters Erzählungen größtenteils eher die textilarmen Interieurs der ersten Jahrhunderthälfte aufrufen, spielt der umfassend präsente Materialkomplex des Textilen (vgl. Bischoff 2005) als „Mittler menschlicher Umweltrelationen“ (Schuster 2014: 307) eine zentrale Rolle. Das Verhältnis von Textilien, Wohnung und Frauenfiguren ist dabei nicht auf eine anthropologisch und mythologisch fundierte Symbolik oder Metaphorik des Weiblichen zu reduzieren. Stattdessen wird in Stifters Texten ein spezifisch weiblich markiertes Stoffwissen verhandelt, dass jedoch in einem nicht zu unterschätzenden Spannungsverhältnis zur männlich markierten Textautorität steht.

Während sich in den Erzählungen Stifters immer wieder männliche Bauherren und Architekten hervortun (von Arburg 2014), liefern zugleich weibliche Figuren die textilen Milieus, in denen sich die Lebensgeschichten als ein Ringen um ein ‚richtiges‘ In-der-Welt-sein abspielen. Im Folgenden gilt es, anhand exemplarischer Schlaglichter auf Stifters Werk das literarisch verhandelte Kräftezerren um die geschlechtliche Autorität in Wohnfragen und die Funktionalität textiler Umgebungen näher in den Blick zu nehmen. Vor diesem Hintergrund lässt sich umgekehrt auch der Einrichtungs‑, Hygiene und Haushaltsdiskurs neu perspektivieren: Wenn sich dort die Zuständigkeit der Frau für das textile Einrichtungswissen als ausgemachte Sache und ideales Modell bürgerlichen Lebens präsentiert, erscheint dies auch als ein Stabilisierungsversuch innerhalb der umfassenden Veränderungen und Verunsicherungen der Wohn- und Lebenszusammenhänge des 19. Jahrhunderts, die die Literatur mit ihren spezifischen Verfahren noch einmal ganz anders in den Blick nehmen kann.

## Literarische Verhandlungen weiblichen Stoffwissens

Mit der weiblichen Handlungsmacht im Interieur verbindet sich für Stifters Erzähler und Figuren ein Moment radikaler Verunsicherung. Das wird besonders deutlich mit Blick auf Erzählungen wie *Das alte Siegel* (1844) oder *Turmalin *(1851), wo geheime Gänge, verdoppelte Interieurs sowie blickdichte Vorhänge und Schleier zur räumlichen Bedingung von Täuschung, Ehebruch und aufgelöster Ordnung werden (vgl. Haag 2012). Vor diesem Hintergrund erklärt sich auch, warum die weibliche Agency in der textilen Wohnausstattung in den meisten Erzählungen Stifters größtenteils gut eingehegt ist.

So entfalten die Erzählungen das weibliche Stoffwissen weniger auf der letztlich männlich dominierten Handlungsebene als vielmehr innerhalb der klaren Grenzen diskursiver Vermittlung. Dazu gehören verschiedene Dialoge zwischen Männer- und Frauenfiguren, in denen letztere in langen Passagen in direkter Rede als Fürsprecherinnen eines textilen Wissens auftreten. Aufgrund der impliziten sexuellen Konnotation wurden diese Gespräche (zum Beispiel in *Der Hagestolz *und *Der Kalkstein*) in der Forschung selten beim Wort genommen und oft auf das dahinter verschleierte bzw. fetischistisch auf den Stoff übertragene Begehren oder das allegorische Textgewebe hin lesbar gemacht (Bischoff 2005; Dangel-Pelloquiun 2008). Dabei gerät leicht aus dem Blick, dass in diesen Passagen durchaus einer zeitgenössischen weiblichen Expertise das Wort gegeben und Stoff als Wissensgegenstand etabliert wird.

In diesem Kontext ist auch an Dithas Flachs-Rede am Ende von *Abdias *(1842) zu denken, in der sie kurz vor ihrem Tod durch einen Blitzschlag eine Art Kulturgeschichte von Flachs und Leinen entwickelt.^12^ Dabei beruft sie sich explizit auf die bisher in der Erzählung nicht näher erwähnte Sara als Quelle dieses weiblich tradierten Wissens, das durch Verben wie ‚wissen‘ und ‚verstehen‘ auch eindeutig als solches gekennzeichnet ist. Dabei macht Ditha das Leinen nicht nur als lebensumspannende Konstante und „eigentlichste Wohnung“ (Stifter 1982a: 340) aus, sondern bettet es in seiner Eigenschaft als Aussteuer oder Arbeitskleidung auch in über das Haus hinausweisende ökonomische und soziale Zusammenhänge ein.

Diese ökonomische Komponente wird in der Erzählung *Der Hagestolz *(1845) besonders deutlich, wenn der junge Victor von seiner Ziehmutter Ludmilla in einer Art Inventur über sein textiles Kapital informiert wird. Sie verweist dabei nicht nur auf die physiologische Funktion des Leinens, das den Körper „schützt und gesund erhält“, sondern rechnet ihm auch genau vor, wie oft er die Kleidung wechseln kann, um den von zu Hause gewohnten hygienischen Standard aufrechtzuerhalten, der als „gelernt“ vorausgesetzt wird und so Teil der von der Ziehmutter vermittelten Bildung ist (Stifter 1982b: 25–26).

Dass den Menschen in der richtigen Anwendung dieses Wissens jedoch nicht ganz zu trauen ist, wird bei Stifter regelrecht zum Motor des Erzählens. So kann – ganz im Sinne der milieutheoretisch geschulten Konzeption von der Wohnung als prägender Umgebung – die falsche Anwendung des textilen Wissens für die charakterliche Bildung der Protagonisten fatale Folgen haben. Das falsche Maß an Stoff bringt in den Erzählungen *Abdias* oder *Der Waldsteig *(1844) defizitäre Charakter hervor, deren Leidens- oder Umbildungswege zum Gegenstand der Erzählung werden.

Im *Waldsteig* ist es die textile Übervorsicht der Mutter, der implizit die Schuld für die Textilneurose des Protagonisten Tiburius zugesprochen wird. Der hypochondrische Tiburius, der „Flanelstreifen auf die Fensterfugen nageln und die Thüren verpolstern“ lässt, schottet sich so durch textile Schutzschichten von der Außenwelt ab (Stifter 1982c: 154). Als Ursache für Tiburius’ Polsterungswut bietet die Erzählung die übertriebene Fürsorge seiner Mutter an, die ihn als Kind stets „warm“ hielt, „daß er sich nicht verkühle“, ihn mit „sehr schöne[n] gestrikte[n] Unterleibchen, Strümpfchen und Ärmlein“ versorgte und eine eigene Strickerin für das Kind beschäftigte (Stifter 1982c: 147). Darin klingt die zeitgenössische, auch in der Medizin geführte Diskussion um Verweichlichung oder Abhärtung an, die in der Frage nach dem textilen Maß konkrete Gestalt gewinnt. In beiden Erzählungen wird dem ‚falschen‘ Umgang mit Textilien eine richtige Textilität entgegengesetzt, wobei dem Leinen als in Stifters gesamtem Werk prominentem Stoff besonderer, ästhetisch, moralisch und medizinisch fundierter Wert zukommt. Bei Tiburius’ schrittweiser Reintegration in die Gesellschaft, die als buchstäblicher Ent- und Auswicklungsprozess inszeniert wird und in einer Heirat kulminiert, handelt es sich letztlich um einen Kleiderwechsel, an dessen Ende der Wandschirm mit „seidenen Chinesen“ und die ledernen „Betten und Lagerstätten“ gegen „bloßes reines[s] Stroh mit Linnendeken“ ausgetauscht werden und Tiburius in einem gut belüfteten Raum in „losen leinenen Kleidern“ geht (Stifter 1982c: 211).

Auch in der ungleich düsterer endenden Erzählung *Abdias *ist der Handlungsverlauf von einem solchen Milieu- bzw. ‚Stoffwechsel‘ geprägt. Die antisemitisch geprägte Erzähllogik legt es implizit nahe, dass der defizitäre Charakter des unter seiner materialistischen Kurzsichtig- und Oberflächlichkeit leidenden sephardischen Juden Abdias auch darauf zurückführen ist, dass ihn seine Mutter Esther als Kind, auf visuelle Effekte berechnet, in Arrangements aus glänzender Seide eingefügt und ihm zum Zeitvertreib verschiedene „Kleidchen“ angelegt oder ihn „als Mädchen“ angekleidet hat (Stifter 1982a: 273).^13^ Auch Esther selbst wiederum wird von Abdias’ Vater Aron gemeinsam mit Abdias zu seinen „höchsten Güter[n]“ (Stifter 1982a: 242) gezählt und in der ägyptischen Höhlenwohnung in einer verfallenen Römerstadt in der Wüste wie ein Schmuckstück im textilen Etui untergebracht.^14^ Und auch der Erzähler macht sich letztlich dieser textilen Fehlbehandlung schuldig, wenn er seine Figuren in textil metaphorisierte Landschaften und Wetterverhältnisse einbindet.^15^

Abdias lässt schließlich den Textilhandel in der nordafrikanischen Welt des Seidenglanzes, der bunten Flecken und Flicken sowie der „verpesteten Lappen“ (Stifter 1982a: 240) und todbringenden Decken (seine Frau stirbt im Kindbett, da ihre Blutungen unter einer Decke verborgen bleiben) hinter sich. Wenn er der neugeborenen Tochter die fehlende Mutterbrust durch eselsmilchgetränkte Leinenstücke ersetzt, ist ein erster Schritt hin zu einer funktionalen Verwendung des Stoffes jenseits von visuellen Effekten getan. Indem Abdias mithilfe dieser textilen Prothese die Mutterrolle übernimmt, wird deutlich, dass die Erzählung Geschlechterrollen weniger festschreibt, als sie vielmehr in ihrer ganzen Dynamik durchspielt. Entsprechend ist die Erzählung im weiteren Verlauf entscheidend von Abdias’ Sorge um die räumliche Einbettung Dithas geprägt, die sich auch immer wieder in der textilen Regulierung ihrer Umwelt niederschlägt (vgl. dazu auch Schuster 2014).

Dithas leinenbasierte Existenz bildet den Ausgangspunkt für ein neues, auf den substantiellen Wert des Leinens gegründetes Leben Abdias’ als Flachsbauer in Österreich. Mit Blick auf die oben dargelegte Befürwortung des Leinens durch Clima und Pettenkofer erscheint Stifters Leinenemphase so nicht allein als nostalgischer Versuch, der im Zuge der Mechanisierung zunehmend von der Baumwolle verdrängten regional verankerten Leinenindustrie ein literarisches Denkmal zu setzen. Die Profilierung des Stoffes lässt sich auch als Auseinandersetzung mit dem materialbezogenen hygienischen Wissen auf der Höhe seiner Zeit lesen und schafft damit wiederum moralische und geographische Besetzungen des Textilen, die auch die medizinischen und materialkundlichen Texte prägen. Mit „verpesteten Lappen“ (Stifter 1982a: 240) einerseits und Tüchern zum Schutz gegen die gefährliche „Wüstenluft“ (Stifter 1982a: 291) andererseits, gibt die Erzählung der ganzen Breite der hygienischen Diskussion um das Textile Raum, ohne sich zwischen den schützenden Eigenschaften von Stoff entsprechend der Miasmentheorie und Konzeptionen des Stoffes als Träger von Kontagien entscheiden zu müssen.^16^

## Gefilterte Schreibräume

Im Gegensatz zu den textil bedingten Entwicklungsstörungen in *Abdias *und *Der Waldsteig* kann die richtige textile Ausstattung wiederum poetologisch wirksame Denk- und Schreibräume eröffnen, wie in der lebenslang überarbeiteten und in einer ersten Fassung 1841 erschienenen Erzählung *Die Mappe meines Urgroßvaters*. Zentrale Funktion kommt dabei Vorhängen für die Gestaltung des erzählten Raumes und als erzählerisches Mittel der bedeutungsproduzierenden Lichtführung und Inszenierung zu. Mit der durch Vorhänge regulierten Raumbeleuchtung wird auf Erzähler- wie auf Figurenebene eine Umgebung geschaffen, die als Denk- und Schreibraum konstitutiv für die als Tagebuchfiktion angelegte Erzählung ist.

Als der Ich-Erzähler der Binnengeschichte, Augustinus, von seinem Medizinstudium in Prag in das Haus seines Vaters zurückkehrt, richten ihm seine Schwestern ein Zimmer her. Über das Bett sind „schneeweiße Tücher […] gespannt, und es harrte auf mich, um in der Nacht meine ermüdeten Glieder aufzunehmen“ (Stifter 1982d: 75). Dazu gibt es einen Tisch, „ebenfalls schneeweiß gescheuert, und auf dem Fußboden knisterte der Sand, den man in der Feuchte einstweilen aufgestreut hatte. Die beiden Fenster waren offen, in dem großen Ofen brannte ein Feuer, damit das ganze Gemach lüfte und trockne“ (Stifter 1982d: 75). In dem gut gelüfteten, mit Feuer beheizten und mit Sand ausgestreutem Raum haben die Schwestern im maßvollen Zusammenspiel der sich gegenseitig regulierenden Elemente ein gesundheitsförderndes Mikroklima der Wohnlichkeit hergestellt.^17^ Der Eindruck von Reinlichkeit wird durch den dominierenden Farbwert des in der gesamten Erzählung präsenten und für Stifters Werk zentralen Weißen (Dangel-Pelloquin 2008; Öhlschläger 2003; Vogel 2003) noch unterstützt. In einem nächsten Schritt erhalten die Fenster des Zimmers noch „zwei sehr schöne Vorhänge, […] die Katharina aus irgend einem schönen Linnen gemacht hatte“ (Stifter 1982d: 76–77):

Nachmittag schien die Sonne recht freundlich in das Gemach, ich zog die Vorhänge zu, wenn ich nach Hause kam, und dann war es dämmerig und lieb um alle Dinge, weil weiße Vorhänge das Licht nicht brechen, sondern blos milder machen; nur daß doch hie und da ein Sonnenstrahl hereinbrach und einen Blitz auf den weißen Boden legte. (Stifter 1982d: 78)

Mit dieser Zimmereinrichtung am Anfang der Aufzeichnungen ist auch der poetologische Rahmen gesetzt, innerhalb dessen sich die Erzählung entfaltet: Die mildernden Vorhänge repräsentieren dabei gleichsam das poetische Surplus des Realismus, mit dem die Wirklichkeit der Dinge in ein bestimmtes Licht getaucht wird. Mit Vorhang, Licht, Dingen und Blitz ist eine Versuchsanordnung gewählt (Schuster 2016: 298), in der sowohl eine klare Grenze zwischen Innen und Außen etabliert sowie deren Überschreitung und Durchdringung inszeniert wird. In der hier vom Vorhangstoff repräsentierten „Paradoxie der Ziehung und Kreuzung der Grenze von Innerem und Äußerem“ wird die Nähe von milieutheoretischen und ästhetischen Diskursen deutlich (Pethes 2017: 153). Mit der Aufmerksamkeit für die „kontingenten externen Faktoren“ der von Hegel als prosaisch abgewerteten Wirklichkeit als „bloße[r] Umwelt des Poetischen“ entwickeln die realistischen Romane und Erzählungen Strategien, um „poetische Effekt[e]“ im „Register des Prosaischen“ zu erzielen (Pethes 2017: 149 f). Als eine solche Strategie lässt sich hier der Vorhang lesen.

Augustinus kann – dank seiner Schwestern – mit den Vorhängen nun die Beleuchtung seiner Umgebung regulieren. Das Gewebe als Kulturprodukt lenkt das von außen eindringende Licht sanft in die richtigen Bahnen und gibt ihm seine spezifische Erscheinungsform im Innenraum (Jürjens 2017). Insofern hat die textile Technologie des Vorhangs ebenso Anteil an der in der Erzählung betriebenen Kultivierung und Besänftigung von Mensch und Natur (Lukas 1996), wie die Straßenbau- und Rodungsarbeiten. Zugleich bleibt dabei im Blitz der unzähmbare Rest an kontingenter Naturgewalt erhalten, an dem sich Stifters Erzählen abarbeitet. Die von den Schwestern bereitgestellten Vorhänge dienen zur Herstellung einer kontemplativen Stimmung, in der Augustinus die schriftliche Nach- und Vorbereitung des vergangenen wie des kommenden Tages vornimmt, wenn er abends „die Vorhänge herab that, und auf dem Papiere anzeigte, was ich heute erfahren habe und was ich morgen unternehmen sollte“ (Stifter 1982d: 135). Das Zimmer wird so zum Denkraum und Ort der literarischen Produktivität, in dem sich in der schriftlichen Beschäftigung mit Vergangenheit und Zukunft die Zeitebenen kreuzen. Scheint das Schreiben hier einerseits auf den Raum als „fruchtbare[r] Matrix“ (Haag 2011: 231) zurück zu führen, ist dieser Raum keineswegs naturgegeben, sondern das Ergebnis ästhetisch und hygienisch informierter Einrichtungsarbeit. Dabei verschränken sich weibliche Einrichtungsexpertise und männliche Autorschaft, wenn die letzte Gestaltungs- und Regulierungsmacht hier im Auf- und Zuziehen in den Händen der männlichen Erzählerfigur Augustinus bleibt.

Ein solcher Prozess der Aneignung textiler Praktiken prägt auch Augustinus’ spätere Errichtung einer Hauskapelle mit doppelt über die Fenster gespannter „mattweiße[r] Seide“, damit „in der Hauskirche so sanfte Dämmerung sei, wie in der großen. – –“ (Stifter 1982d: 198) Dass das Vorbild für diesen Raum weniger die große Kirche, als vielmehr Margarita zu sein scheint, deutet sich in den doppelten Gedankenstrichen nach der Auslassung an. Mit diesem Bauprojekt errichtet er seiner stets weißgekleideten großen Liebe Margarita eine Art räumliches Denkmal und Substitut, nachdem er sich in Folge eines Eifersuchtsausbruchs mit ihr überworfen hat. Und auch die blauen und roten Seidenbänder mit denen Margarita ihre Kleider verziert, werden von Augustinus übernommen, wenn dieser sein Tagebuch – entsprechend der von Margaritas Vater angeratenen Strategie – in einzelnen Abschnitten mit den Bändern versiegelt (vgl. dazu Schneider 2008: 173). Wenn dieses Tagebuch wiederum im Sinne der in der Rahmenerzählung etablierten Archivfiktion uns als Lesenden vorliegt, erweisen sich weibliche Textil- und männliche Textpraxis bei Stifter als eng verflochtene Einheit.

In den hier stichprobenartig untersuchten literarischen Beispielen zeigt sich, wie tief Stoffe in Stifters Erzählwelten eingreifen und dass sie existenzielle und entwicklungsrelevante Milieus für die Figuren wie für das Erzählen selbst bereitstellen. Auch wenn die von den Frauenfiguren eingesetzten Textilien dabei vor allem ermöglichende Strukturen für die Bildungsgeschichten der männlichen Protagonisten und Erzählerfiguren bereitstellen (vgl. dazu den Beitrag von Susanne Schmidt), lässt sich mit ihnen zugleich ein marginalisiertes weiblich konnotiertes Wissen sichtbar machen. Dieses bleibt allerdings in den Bereich der Rede eingehegt oder wird letztlich von den männlichen (Erzähler‑)Figuren übernommen. Weniger als eine klare geschlechtergemäße Zuordnung und Befriedung von Wohn- und Einrichtungsfragen, erscheinen Stifters Erzählungen so als Verhandlungen der damit verbundenen Spannungen.

## Maschinistin des Lebens

Die Frage des textilen Maßes und des richtigen Einsatzes von Einrichtungsstoffen prägt im 19. Jahrhundert die hygienische, ästhetische und literarische Diskussion um das richtige Wohnen. Dabei zeigt sich, dass nicht allein der Einfluss einer volkserzieherischen Hygiene zur Verwissenschaftlichung der Hausarbeit führt, sondern dass auch in den ästhetisch ausgerichteten und literarischen Texten die Technologien des Wohnens und Einrichtens als lehr- und lernbare Wissensgegenstände verhandelt werden. Als „weiteres Kleid“ (Falke 1873: 2) steht die Wohnung in engem Bezug und Austausch zu den darin lebenden Menschen und wird als ästhetisch bildende Umgebung und Voraussetzung von Gesundheit entworfen. Häusliche Textilien erscheinen in dieser Logik als Erweiterungen des Körpers, die den Austausch mit der Umgebung regulieren und insofern wiederum auf die Bewohnenden einwirken. Vor diesem Hintergrund sind die Textilien im Interieur des 19. Jahrhunderts nicht allein Verkleidungen im Theater der Domestizität (vgl. Meyer 1982) oder Mittel, um „ein künstliches Märchenreich in die Eintönigkeit des Industriealltags“ zu zaubern (Giedion 1982 [1948]: 403). Vielmehr gewinnen sie für das Wechselverhältnis von Lebewesen und Umgebung durchaus existentielle Bedeutung. Damit kommt der mit der textilen Ausstattung betrauten Frau entscheidende Verantwortung zu, deren Wirkbereich über die durchlässig entworfenen Grenzen des Hauses hinausgeht.

Im Zuge der Verwissenschaftlichung des Wohnens zerlegen die Hygieniker wie Pettenkofer die Wohnung in quantifizierbare und in Versuchsreihen genauestens untersuchte Einzelteile (vgl. Ekici 2017), die Haushaltslehrbücher unterteilen die Hausarbeit nach Räumen, Tageszeiten sowie Arbeitsschritten und die ästhetischen Einrichtungsratgeber widmen sich einzelnen Räumen, Raumelementen, Materialien und Möbelstücken. Auch die Literatur verhandelt, wie am Beispiel Stifters gezeigt wurde, in regelrechten Experimentalanordnungen das Verhältnis von Lebewesen und Umgebung. Diese jeweils ganz unterschiedlich motivierten, analytischen oder narrativen Bemühungen, zielen letztlich darauf die physiologische Einheit von Körper und Wohnung zu fassen, zu optimieren oder zu evozieren. Dabei lassen sich drei – zugleich eng miteinander verflochtene – Tendenzen in der Auseinandersetzung mit der häuslich-textilen Umgebung ausmachen: Sie wird (1.) als Nest und Haut naturalisiert und feminisiert, (2.) als Apparat und Maschine technisiert sowie (3.) als Lebensgrundlage und Filter poetisiert.

Vor diesem Hintergrund lässt sich die Vorstellung von der Frau im Interieur des 19. Jahrhunderts womöglich etwas modifizieren: Statt einer dem Interieur einverleibten und darin zu Leblosigkeit erstarrten Zierde des Hauses zeichnet sich ein Bild der Frau als Maschinistin des Lebens ab. In diesem Sinne scheint es wegweisend, wenn die Autorin Helene Böhlau in ihrem 1899 erschienenen Roman *Halbtier! *eine Auseinandersetzung des Ehepaars Frey in maschineller Metaphorik beschreibt: „Die Frau streifte ihn [den Ehemann, K.J.] mit einem einzigen langen Blick, wie ein Heizer etwa auf das Ventil seiner Dampfmaschine schaut, mit unendlicher Sachkenntnis“ (Böhlau 2020: 24). Vor der Unsichtbarkeit schützt allerdings auch die Verwissenschaftlichung und Technisierung des Interieurs in letzter Konsequenz nicht: Mit der Maschine ist ein zentraler Topos des Umweltdenkens angesprochen, dem zugleich das Problem innewohnt, dass in der wechselseitigen Verbindung kaum zwischen „Maschine“ und „Maschinist“ zu unterscheiden ist (Canguilhem 2009: 255). Erst mit der Umwelttheorie Jakob von Uexkülls um die Jahrhundertwende wird die Aktivität des Lebewesens betont, das nach Canguilhem „keine Maschine“, sondern „ein Maschinist“ ist, „der mit Handlungen auf Signale reagiert“ (Canguilhem 2009: 262). Umso wichtiger ist es, die von der Maschinen-Metapher suggerierte passive Automatik, die auch die medizinische Vorstellung des Frauenkörpers entscheidend geprägt hat (Martin 2001), zu hinterfragen, und stattdessen die Arbeit der Maschinistinnen bzw. die den Milieu- und Umwelttheorien eingeschriebenen geschlechtlichen Besetzungen und Verhandlungen sichtbar zu machen.

## Footnotes

1 In Walter Benjamins in der Textsammlung *Einbahnstraße* erschienener Miniatur *Hochherrschaftlich möblierte Zehnzimmerwohnung* heißt es entsprechend: „Das bürgerliche Interieur der sechziger bis neunziger Jahre […] wird adäquat nur der Leiche zur Behausung. Auf diesem Sofa kann die Tante nur ermordet werden. Die seelenlose Üppigkeit des Mobiliars wird wahrhafter Komfort erst vor dem Leichnam“ (Benjamin 1972: 89).

2 So beschreibt beispielsweise Vicki Baum wie sie und die „Mädchen von 1905“ im Gegensatz zu den „Mütter[n] von 1890“ die „Eierschalen des neunzehnten Jahrhunderts nach Möglichkeit abgestreift“ hätten und voller „Hunger nach wirklichem Leben ohne Fiktionen und Verschleierungen“ seien (Baum 1988: 32).

3 Für entscheidende Hinweise und Impulse zu einer frühen Vortragsfassung dieses Artikels danke ich Christine von Oertzen.

4 Bei Fonssagrives heißt es im französischen Original: „L’homme peut être considéré comme placé au milieu d’une succession de sphères concentriques, dont le rayon va s’agrandissant de plus en plus à mesure, qu’elles s’éloignent de lui, elles représentent la série des influences physiques qu’il subit, tout en les modifiant à son gré: le vêtement, la maison, la ville, le climat“ (Fonssagrives 1871: 1).

5 Ganz ähnlich heißt es auch bei Fonssagrives: „La femme, pour qui cette chambre figure, sous des proportions réduites, le gynécée antique, est chez elle dans sa chambre à coucher; elle doit s’y plaire et s’y trouver à l’aise. Il faut donc qu’elle la choisisse bien, et qu’elle l’installe à son gré. C’est, en effet, la petite capitale, de ce royaume pacifique du ménage, le propre domaine“ (Fonssagrives 1871: 101).

6 In diesem Zusammenhang ist auch Jakob von Falkes Aufforderung zu betrachten, ihre „Toilette“ nicht zu vernachlässigen, um nicht etwa „in ihrer kunstreich geschmückten Umgebung eine Disharmonie [zu] sein und das Bild, das sie selber geschaffen hat, durch ihre eigene Person, durch den Contrast, den sie darstellt, wieder zerstören.“ (Falke 1873: 353) Ähnlich heißt es auch bei Gurlitt: „Die Färbung von Wänden und Möbeln richte sich nach den persönlichen Wünschen der Besitzerin, sei ihrem Hauttone, ihrem Haar, den bevorzugten Farben ihrer Kleidung angepasst.“ (Gurlitt 1888: 108).

7 An der Diskussion um Abhärtung oder Verweichlichung beteiligt sich in der *Gartenlaube *besonders der Anatom Carl Ernst Bock, der sich explizit für Textilien als Schutz vor Krankheiten ausspricht und dabei die Bauchbinde zum Schutz vor der Cholera empfiehlt: „Man lasse sich’s doch gesagt sein, daß durch ‚Warmhalten des Bauches‘ der Ausbruch der Cholera verhütet werden kann“ (Bock 1866: 623).

8 Ute Frevert zeigt, dass die vom Bürgertum ausgehende „Hygienisierung des Familienlebens“ die Arbeiterfrauen einem enormen Leistungsdruck aussetzte, „der ihre Arbeitskraft permanent überbeanspruchte“ (Frevert 1985: 435).

9 Die Vorstellung, dass das Bett wie die Kleidung die Belüftung zur Wärmeregulation leisten müsse, ist in Schriften zur Haushaltsgesundheit verbreitet. So beschreibt Anna Fischer-Dückelmann das Bett als „Kleidung für die Nacht“, die „Außenluft eindringen“ und „Innenluft heraustreten lassen“ müsse (Fischer-Dückelmann 1911: 163). Ähnlich spricht auch Friedrich Dornblüth vom Bett als „Kleid für die Nacht“ (Dornblüth 1878: 426) und „Nachtkleid“ (Dornblüth 1878: 427), das „die Körperwärme zusammenhalten [soll], ohne uns zu überhitzen und ohne die Lüftung unserer Hautfläche zu verhindern“ (Dornblüth 1878: 427).

10 Didem Ekici weist auf den durchaus paradoxen Charakter hin, mit dem die zunehmende Mechanisierung des Hauses dieses zugleich mehr und mehr dem anatomischen Körper angleicht (Ekici 2017).

11 Im Folgenden beziehe ich mich nicht auf die als Journalfassungen bekannten Erstveröffentlichungen, sondern auf die überarbeiteten und zwischen 1844 und 1850 in mehrbändiger Buchform als *Studien *erschienenen Zweitfassungen der Texte.

12 In der Erzählung heißt es: „[E]s hat mir Sara auf mein Befragen vor langer Zeit, […], vieles von dem Flachse erzählt, aber ich habe es damals nicht verstanden. Jetzt aber verstehe ich es und habe es selbst beobachtet. Sie ist ein Freund des Menschen, diese Pflanze, hat Sara gesagt, sie hat den Menschen lieb. Ich weiß es jetzt, daß es so ist. Zuerst hat sie die schöne Blüthe auf den grünen Säulchen, dann, wenn sie todt ist, und durch die Luft und das Wasser zubereitet wird, gibt sie uns die weichen silbergrauen Fasern, aus denen die Menschen die Gewebe machen, welches, wie schon Sara sagte, ihre eigentlichste Wohnung ist, von der Wiege zum Grabe. Siehst du, das ist auch wahr: – wie sie nur so wunderbar, diese Pflanze zu dem weißen lichten Schnee zu bleichen ist – dann legt man die Kinder, wenn sie recht klein sind, wie ich war, hinein und hüllt die Glieder zu – ihrer Tochter hat Sara viel Linnen mit gegeben, da sie fort zog, um den fremden Mann zu heirathen, der sie wollte; sie war eine Braut, und je größere Berge dieses Schnee’s man einer Braut mitgeben kann, desto reicher ist sie – unsere Knechte tragen die weißen Linnenärmel auf den bloßen Armen – und wenn wir todt sind, hüllen sie die weißen Tücher um uns, weißt du“ (Stifter 1982a: 340).

13 In der Erzählung heißt es, dass Esther Abdias in die „mit Seide ausgefüttert[e] und mit seidenen Polstern bedeckt[e]“ Mauernische legt, „damit sich sein süßes Lächeln recht heiter von der schönen dunklen grünen Seide hervorhebe“ (Stifter 1982a: 273). Dass sich mit der Charakterisierung und der impliziten Feminisierung Abdias’ auch antisemitische Klischees verbinden, hat die Forschung hervorgehoben (Hoffmann 2011).

14 Das erste, nach Esther benannte Kapitel der Erzählung führt diese über ein textiles Arrangement ein: „Ihr Leib ruhete auf dem Seidengewebe von Damaskus und ihre Schultern wurden geschmeichelt von dem weichsten und glühendsten aller Zeuge, dem gewebten Märchen aus Kaschemir, so wie es auch die Sultana in Stambul hat.“ (Stifter 1982a: 242)

15 Mit der grundlegenden Bedeutung, die Textilien in der Erzählung für die Herstellung von bewohnbaren Lebensräumen zukommt, greift das Textile auf der Sprachebene auch auf atmosphärische und landschaftliche Phänomene über, wenn die Wolken wiederholt als „Ballen“ (Stifter 1982a: 284) beschrieben werden oder der Sonnenaufgang, als der Zeitpunkt, wenn „sich der erste Saum des leeren Himmels in Osten anzündete“ (Stifter 1982a: 290).

16 Als Vertreter einer solchen Zwischenposition kann auch der Anatom und Pathologe Jakob Henle gelten, der in seiner Schrift *Miasmen und Kontagien *(1840) auf die Gefahr von Textilien als Träger von Krankheitserregern hinweist. Die „besten Träger“ des „luftförmig ausgeschieden[en] oder mit seinen organischen Vehikeln haft[enden]“ Kontagiums seien „bekanntlich feine, poröse, tierische oder pflanzliche Teile, Wolle, Haare, Federn, Hörner, Häute, Holz, Leinwand, Papier“ (Henle 1910 [1840]: 18 f).

17 Die Notwendigkeit, das Mauerwerk von Neubauten oder wenig bewohnten Räumen trocknen zu lassen, wird in der Erzählung mehrfach erwähnt und beschäftigt auch die Hygiene des 19. Jahrhunderts. Während die von den Schwestern angewandte Kombination aus Heizen und Lüften noch 1872 von Max von Pettenkofer in seinen *Populären Vorlesungen* als das „einzige rationelle, aber zugleich auch das sicherste Mittel“ beschrieben wird, „um Neubauten rasch zu trocknen“ (Pettenkofer 1873: 55), wendet Augustinus später in seinem Neubau mit Pottasche, „die wir vorher glühend gemacht und wieder abgekühlt hatten, damit sie die feuchten Dünste, die aus der Mauer kamen, einsaugen möchte“ (Stifter 1982d : 91), ein Mittel an, das Pettenkofer als „blosse Täuschung“ abwertet (Pettenkofer 1873: 55).
